# Localization of hypnopompic seizures – A stereo EEG study

**DOI:** 10.1016/j.ebr.2024.100729

**Published:** 2024-11-19

**Authors:** Mohammad Alisali, Stuti Joshi, Chaitanya Ganne, Vladimir Vashin, Sandipan Pati

**Affiliations:** Department of Neurology, McGovern Medical School, University of Texas Health Science Center at Houston, TX, USA

**Keywords:** Hypnopompic seizures, Arousal, Sleep, Stereo EEG, EMU

## Abstract

Hypnopompic seizures, characterized by arousal from sleep as the primary clinical manifestation, are a rare and challenging seizure type. Their exact localization has been elusive, often requiring stereotactic EEG (SEEG) for accurate identification. We present the case of a 23-year-old male with drug-resistant focal epilepsy, in whom hypnopompic seizures were localized to the mesial orbitofrontal cortex, with rapid recruitment of the middle temporal gyrus, fusiform gyrus, rostral cingulate, and amygdala. SEEG captured multiple seizures, with arousal occurring 4–5 s after EEG onset, followed by ictal central apnea. The patient underwent resection of the right orbitofrontal and mesial temporal lobe regions and has remained seizure-free for over seven months. This case provides new insights into the neuroanatomical origins of seizure-induced arousal, identifying the mesial orbitofrontal cortex as a potential site for hypnopompic seizures. The findings underscore the critical role of SEEG in accurately localizing seizure foci in complex epilepsy cases, enabling targeted surgical interventions. Additionally, we discuss the orbitofrontal cortex’s role as a site for extra-thalamic arousal pathways, offering new perspectives on the mechanisms underlying hypnopompic seizures.

## Highlights

• Hypnopompic seizures are a rare and challenging seizure type.
• Scalp EEG is usually of limited value for their localization.
• Stereo EEG localizes hypnopompic seizures to the mesial orbitofrontal cortex.
• Mesial frontal lobe structures may represent an extra thalamic arousal system.

## 1 Introduction

The relationship between sleep and epilepsy is well-established and complex. Many sleep-related seizures are associated with arousal. However, it is unclear whether the change in brain state (i.e., arousal) facilitates seizure onset or if the seizure itself induces arousal. Awad and Lüders coined the term “hypnopompic seizure” to describe seizures where arousal from sleep is the only or most prominent clinical manifestation [1]. They reported this phenomenon in a case series of five patients who underwent scalp EEG investigation. Despite these findings, the precise localization of hypnopompic seizures remains elusive, typically requiring stereotactic EEG (SEEG) for accurate identification. Here, we describe a case of hypnopompic seizures localized to the mesial orbitofrontal cortex using SEEG, providing new insights into the localization of this rare seizure type.

## 2 Case report

The patient is a 23-year-old right-handed male with drug-resistant focal epilepsy since age 19. His diagnosis was delayed due to initial presentations of waking up from sleep with psychosis. The epilepsy diagnosis was confirmed after a witnessed convulsive seizure during sleep led to a video EEG investigation. The patient reported two types of seizures: a) Focal impaired awareness seizures: These occurred monthly (1–2 times) and involved arousal from sleep, progression to automotor activity, and eventual postictal psychosis. B) Focal to bilateral tonic-clonic seizures: These occurred occasionally (once a year) and were followed by postictal psychosis.

**Fig. 1A:**
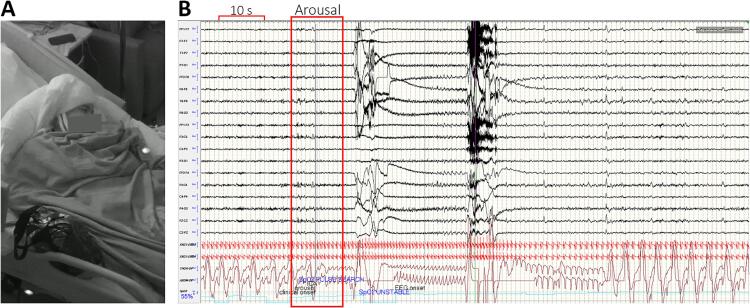
Video of the hypnopompic seizure. 20 s into the video, the patient arouses and opens their eyes. B: Simultaneous scalp EEG shows that the clinical onset (arousal with eyes opening) preceded the EEG onset (rhythmic alpha activity in the right frontotemporal region) by about 20 s. Note the thoracic belts indicating apnea preceding the EEG onset.

Video scalp EEG revealed a normal interictal recording. During five days of scalp EEG study, the patient had two hypnopompic seizures with subtle EEG changes. During these seizures, there was questionable rhythmic alpha activity in the right frontotemporal region, with some evolution in morphology, lasting about 30 s (Figs. 1A-B). The only clinical manifestation was arousal from sleep, followed by ictal central apnea detected with pulse oximetry and thoracic and abdominal belt sensors. Clinical onset preceded the scalp EEG changes by about 20 s.

A 3 Tesla MRI brain scan did not reveal an epileptogenic lesion. A fludeoxyglucose positron emission tomography (PET) showed mild hypometabolism in the right mesial/anterior temporal lobe. Magnetoencephalography (MEG) revealed frequent discharges in loose clusters within the right anterior and mid-temporal regions. Neuropsychological evaluation showed non-dominant frontotemporal dysfunction with some involvement of mesial temporal structures.Based on these results, the hypothesis proposed during the patient management conference was that the putative epileptogenic network involved the right mesial temporal lobe or a temporal-plus region with potential orbitofrontal involvement. SEEG was recommended to localize the seizure focus.

The rationale for mesial temporal lobe epilepsy included the presence of ictal central apnea, typically localized to the amygdala, and the MEG findings. The orbitofrontal region was suspected due to the presence of arousal, scalp EEG findings, and known connectivity to mesial temporal lobe structures.

**Fig. 2A:**
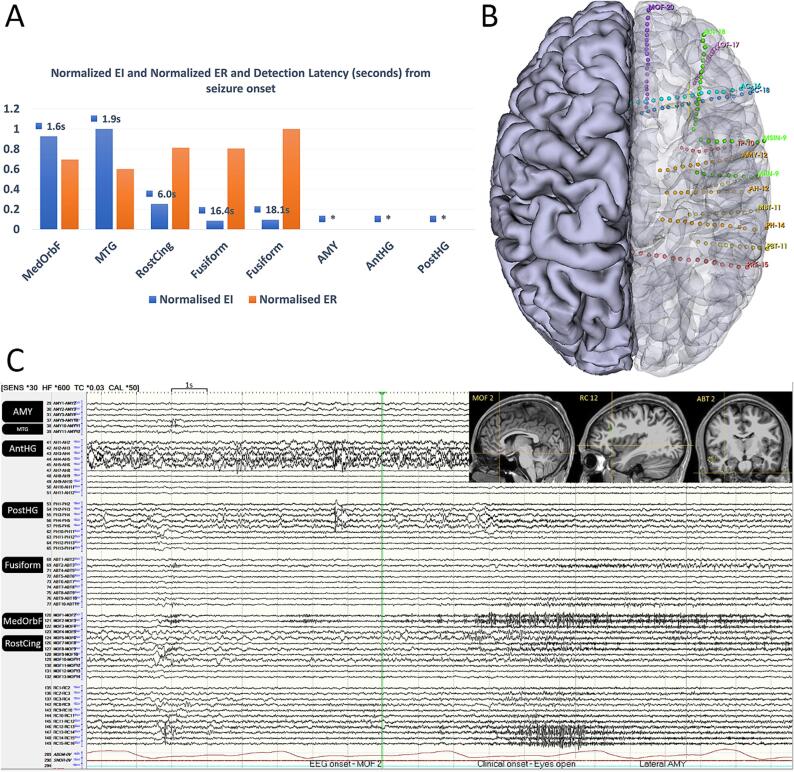
Epileptogenicity Index (EI), Energy Ratio (ER), and detection latency of the involved brain regions at seizure onset and early spread. B: Unilateral (right hemisphere) SEEG implant strategy. C: SEEG showing seizure onset with low voltage fast activity (LVFA) in the medial orbitofrontal cortex (MOF 1–4). Abbreviations: MedoRbFr—medial orbitofrontal cortex, MTG—mesial temporal gyrus, RostCing—rostral cingulate, AMY—amygdala, AntHg—anterior hippocampal gyrus, PHG—posterior hippocampal gyrus.

During the SEEG investigation, 14 electrodes were implanted targeting the right mesial and lateral temporal regions, orbitofrontal cortex, anterior and posterior insular structures, anterior cingulate, and precuneus (Fig. 2B). The SEEG revealed two interictal populations: a) Right hippocampus (AH 2–5 and pH 2–5): spikes and continuous slowing with admixed fast activity. B) Right mesial orbitofrontal cortex (MOF 2): bursts of gamma activity occurring independently or synchronously with the anterior insula/frontal operculum (RC 11–15), lateral temporal (AMY 10–12), and basal temporal (ABT 2–3) regions.

Six seizures were captured, all clinically identical to those recorded during the scalp EEG study, manifesting as arousal and ictal central apnea. Only one seizure progressed to impaired awareness. The seizures began with low-voltage fast activity (LVFA) in the medial orbitofrontal cortex (MOF 2–3), rapidly propagating to the anterior insula and frontal operculum (MOF 6–8, RC 11–15), fusiform gyrus (ABT 2–3), middle temporal gyrus (AMY 10–12), and later spreading to the hippocampus (AH 2–5 and pH 2–5) (Fig. 2C). The ictal onset preceded the clinical onset (arousal) by an average of 4–5 s. We quantified the seizure onset zone using the epileptogenicity index EI [2] with Anywave software [3]. The EI is a semi-automated method for quantifying the epileptogenicity of various brain structures using SEEG signals and is not intended as a seizure detection algorithm. The medial orbitofrontal cortex and the middle temporal gyrus showed the highest EI with shorter detection latency for the medial orbitofrontal cortex (Fig. 2A). Direct electrical stimulation of the orbitofrontal region at 50 Hz during wakefulness induced a 12-second run of after discharges without apparent clinical or behavioral changes. Stimulation of the hippocampus at 1 Hz induced after discharges, while stimulation of the amygdala at 50 Hz induced central apnea.

Based on these findings, the multidisciplinary epilepsy surgery conference concluded that the epileptogenic network included the right orbitofrontal and mesial temporal lobe regions. The patient was offered a right mesial orbitofrontal resection combined with a right anterior temporal lobectomy. Histopathology demonstrated gliosis and chronic inflammatory changes. The patient remains seizure-free eight months post-resection. An informed consent was obtained from the patient.

## 3 Discussion

Sleep arousals are defined as transient changes in brain state from sleep to wakefulness or during transitions between sleep stages, such as rapid eye movement (REM) and non-rapid eye movement (NREM). The temporal relationship between seizures and arousal is complex; some seizures are facilitated by arousal, while others evoke an arousal response. Malow et al. [4], using combined scalp-intracranial electrodes, demonstrated that most sleep-related temporal lobe seizures occur during NREM sleep and precede arousals, with seizures provoked by arousal being the exception. Hypnopompic seizures are distinct in that they induce arousal, with arousal being the sole behavioral manifestation of the seizure. This uniqueness makes diagnosing hypnopompic seizures challenging, as seen in our patient, often requiring video EEG (VEEG) and polysomnography for confirmation. The differential diagnosis for confusional arousal includes conditions such as obstructive sleep apnea (OSA) and NREM sleep parasomnias like sleep terrors and sleepwalking. Each of these conditions shares features with hypnopompic seizures but requires distinct diagnostic and therapeutic approaches. Therefore, accurately distinguishing between these conditions with VEEG is crucial for effective patient management.

Intracranial EEG studies of hypnopompic seizures are scarce. Previous studies have implicated structures in the frontal lobe as the symptomatogenic zones [5], [6]. In our case, using SEEG and epileptogenicity index (EI), we localized the hypnopompic seizures to the right mesial orbitofrontal cortex with rapid recruitment of the rostral cingulate, fusiform cortex, and temporal lobe. This precise localization allowed for a targeted surgical intervention, which significantly improved the patient's outcome.

The mechanisms of physiological arousal are well understood, involving a bipartite arousal system: a cholinergic system originating in the pedunculopontine (PPT) and laterodorsal (LDT) tegmental nuclei that projects to the thalamic midline and intralaminar nuclei; and a monoaminergic system that bypasses the thalamus to activate neurons in the hypothalamus, basal forebrain, and cortex directly [7]. However, the exact pathophysiology of ictal arousal remains unknown. It is known that ictal spread to the thalamus and other subcortical regions can cause impaired awareness during focal seizures [8], suggesting a similar mechanism might underlie ictal arousal.

The orbitofrontal cortex plays a crucial role in processing rewards and punishments [8], receiving input from all sensory modalities through reciprocal connections with cortical, thalamic, and hypothalamic regions. Notably, the orbitofrontal cortex receives cholinergic input from the nucleus basalis of Meynert in the basal forebrain and projects back to it, thereby potentially controlling cholinergic input to the entire cerebral cortex [9]. Based on both basic science and clinical data, Mashour et al. [10] have proposed the medial prefrontal cortex as a critical node within arousal-promoting networks. Therefore, the localization of hypnopompic seizures to the medial orbitofrontal cortex in our case and to other frontal lobe structures mainly the anterior cingulate in the previous report may reflect ictal activation of an extra-thalamic arousal network.

Further research is needed to explore other cortical regions potentially involved in ictal arousal and better understand this phenomenon's underlying mechanisms.

## CRediT authorship contribution statement

**Mohammad Alisali:** Writing – original draft, Resources, Data curation. **Stuti Joshi:** Data curation. **Chaitanya Ganne:** Software. **Vladimir Vashin:** Visualization. **Sandipan Pati:** Writing – review & editing, Supervision, Conceptualization.

## Declaration of competing interest

The authors declare that they have no known competing financial interests or personal relationships that could have appeared to influence the work reported in this paper.
